# Economic Challenges in Nutritional Management

**DOI:** 10.3390/jcm8071005

**Published:** 2019-07-10

**Authors:** Emilie Reber, Kristina Norman, Olga Endrich, Philipp Schuetz, Andreas Frei, Zeno Stanga

**Affiliations:** 1 Department for Diabetes, Endocrinology, Nutritional Medicine and Metabolism, Bern University Hospital, and University of Bern, 3010 Bern, Switzerland; 2 Department of Nutrition and Gerontology, German Institute for Human Nutrition Potsdam-Rehbrücke, 14558 Nuthetal, Germany; 3 Research Group on Geriatrics, Charité Universitätsmedizin Berlin, Corporate Member of Freie Universität Berlin, Humboldt-Universität zu Berlin, and Berlin Institute of Health, 13347 Berlin, Germany; 4 Health Data Management and Health Economics, Medical Directorate, Bern University Hospital, and University of Bern, 3010 Bern, Switzerland; 5 Medical University Department, Division of General Internal and Emergency Medicine, Kantonsspital Aarau, 5001 Aarau, Switzerland; 6 Freelance Health Economist, 4133 Pratteln, Switzerland

**Keywords:** economic challenges, nutritional management, malnutrition

## Abstract

Disease-related malnutrition (DRM) is a highly prevalent independent risk and cost factor with significant influence on mortality, morbidity, length of hospital stay (LOS), functional impairment and quality of life. The aim of our research was to estimate the economic impact of the introduction of routinely performed nutritional screening (NS) in a tertiary hospital, with subsequent nutritional interventions (NI) in patients with potential or manifest DRM. Economic impact analysis of natural detection of inpatients at risk and estimation of the change in economic activity after the implementation of a systematic NS were performed. The reference population for natural detection of DRM is about 20,000 inpatients per year. Based on current data, DRM prevalence is estimated at 20%, so 4000 patients with potential and manifest DRM should be detected. The NI costs were estimated at CHF 0.693 million, with savings of CHF 1.582 million (LOS reduction) and CHF 0.806 million in additional revenue (SwissDRG system). Thus, the introduction of routine NS generates additional costs of CHF 1.181 million that are compensated by additional savings of CHF 2.043 million and an excess in additional revenue of CHF 2.071 million. NS with subsequent adequate nutritional intervention shows an economic potential for hospitals.

## 1. Introduction

Disease-related malnutrition (DRM) is a debilitating, important and frequently occurring problem with an estimated prevalence of 20–50% on hospital admission [1,2,3,4,5]. The consequences of DRM are well known: Increases in morbidity, complication and mortality rates, resource use for inpatient treatment, prolonged hospital length of stay (LOS), decreased quality of life (QoL) and decreased body function. Many studies have shown the positive effects of nutritional interventions (NI), mainly in the reduction of complication rates, LOS, or rates of non-elective re-hospitalizations [3,6,7,8,9,10]. The recently published study by Schuetz et al.—a multicenter study with 2088 medical patients at nutritional risk, from eight Swiss hospitals—showed a significant reduction in serious complications and mortality as well as an improvement in physical function and quality of life after 30 days [11]. The study by Deutz et al. also showed a significant reduction in mortality after 90 days [12]. Available studies on cost-effectiveness clearly show that NI can save money and that the costs of NI are more than offset by the savings [7,9,13].

Since 2012, hospitals in Switzerland are remunerated using per-case rates for inpatients through the Swiss Diagnosis Related Groups (SwissDRG) system. In the SwissDRG it is possible to take DRM into account by coding it as a principal or secondary diagnosis. This requires optimal nutritional management from the hospitals, with an initial screening and adequate therapy in the course of treatment. Coding DRM may have the effect that a certain percentage of the patients will be assigned to a DRG with a higher cost-weight, generating additional revenue for the hospital (like a bonus system). Aeberhard et al. investigated the financial effects of coding DRM in the SwissDRG system, including all inpatients from the years 2013 to 2016 in our university hospital. During the observation period, 3.2% of the patients were coded with DRM. In 8.3% of these cases, the coding led to the attribution of a DRG with an increased cost-weight. This resulted in total additional revenue of circa CHF 3.5 million, which was offset by costs of CHF 2.8 million for assessment and treatment of DRM [1]. Thus, the costs of screening and treatment of DRM were already overcompensated for as a result of the DRM coding and changes in SwissDRG attribution. These results have been confirmed in similar studies [14,15].

As regularly performed nutritional risk screening is not established at the Bern University Hospital, these results reflect the natural detection (ND) of DRM. If nutritional screening (NS) is performed routinely on all patients, the detection rate of DRM will likely increase, leading to earlier detection and treatment. Performing routine screening will generate further costs. Besides that, the same costs for each patient at nutritional risk occurs when the NS is performed, as in the natural detection of DRM. However, due to an increased detection rate, the number of patients with treated and coded DRM will rise, with NI costs increasing as a consequence, but with additional revenues generated [16,17]. If DRM is detected and adequately treated, three types of consequences will occur:

- There will be costs for the detection of DRM and consequently for nutritional treatment,
- Treatment of DRM will lead to improvements in clinical outcomes that transfer to savings in the costs of basic treatment, and
- Patients may be attributed to DRGs with increased cost-weights, causing additional revenues for the hospital.

Previous economic analyses have always compared either the costs of NI with savings in the costs of basic nutritional treatments [3,7,13] or with the additional revenues due to DRG changes [14,16,17]. No study is known to have compared costs with a combination of both savings and additional revenue.

The objective of this study was to estimate the economic impact of the introduction of a systematically performed NS on all patients in our hospital, with subsequent NI in all patients at nutritional risk.

## 2. Materials and Methods

**Figure 1:**
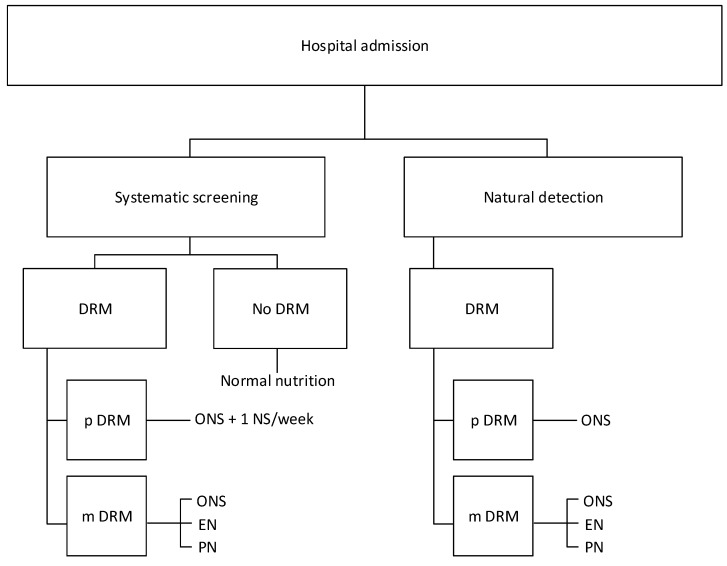
Nutritional screening (NS) and natural detection (ND). DRM: Disease-related malnutrition; p DRM: potential DRM; m DRM: manifest DRM; ONS: oral nutritional supplements; EN: enteral nutrition; PN: parenteral nutrition.

The data for this economic impact analysis were collected from the electronic patient record system of the Bern University Hospital in Bern, Switzerland, between 1 January and 31 December 2018. This is a cost-minimization analysis of the short-term economic consequences of inpatient hospitalization for the year 2018. Included were direct medical costs for NS and NI, cost savings due to NI, and additional revenues due to DRM coding and DRG changes. Using a population-based approach, the economic effects of a systematically performed NS were projected and compared with the existing situation reflecting the natural detection of DRM and routine nutritional management. To this end, a flow chart was created, and the patient flows and resource use were assessed. The costs of ND were subtracted from those of a systematic NS. Furthermore, the number of additional staff needed to perform routine NS was estimated. Data were obtained through analyses of the literature, the use of secondary statistics, and data collection at the Bern University Hospital. In cases of unclear or controversial data, assumptions were made. The flow chart is presented in Figure 1.

In a systematic NS, all patients are screened on admission to the hospital using a validated tool—in our case, the Nutritional Risk Screening 2002 (NRS 2002) [18]. Patients with an NRS score of 2 points or less are likely to be unaffected. Patients with an NRS score of 3 are considered at risk of DRM, and those with a score >3 points are considered to have manifest DRM. Patients with potential DRM undergo NS once per week. Patients with potential DRM will receive oral nutritional supplements (ONS) in addition to the customary nutrition and will also undergo NS once per week. Patients with manifest DRM will receive specific NI such as ONS, enteral nutrition (EN), or parenteral nutrition (PN) as needed and indicated in addition to the customary hospital nutrition. Their nutritional status will be regularly observed so that in these patients no additional NS will be needed [19].

## 3. Input Data

In Table 1 the input data are summarized.

### 3.1. Target Population

For a systematic NS with subsequent NI, patients with an expected LOS of >3 days were considered eligible. In 2018, this amounted to 21,819 patients (information from Bern University Hospital). The average LOS of these patients was 10.01 days. This number also included healthy newborns. Therefore, it was assumed that the target population for a systematic NS at the Bern University Hospital would amount to approximately 20,000 patients yearly. Even though Aeberhard et al. [1] included all inpatients, it is unlikely that DRM was detected in patients with a LOS ≤3. Therefore, the reference population for ND patients with DRM was also assumed to be about 20,000 patients per year.

### 3.2. Detection Rate

Detection rates of 19% and 25.6% were obtained from two German studies [16,17]. The latter, however, also included patients with NRS ≥2, and the rate of patients with NRS >3 was computed at 20.7%. A Dutch study found that NS could increase the detection rate of DRM from 50% to 80% given a DRM prevalence of 32% [20]. Based on these data, the detection rate was estimated at 20%. For natural detection, we doubled the rate of 3.2% from Aeberhard et al. [1], using 6.4%.

### 3.3. Proportions with Potential and Manifest DRM

In patients with NS, the proportion of potential versus manifest DRM was estimated at 45%:55% based on [17]. In the naturally detected patients, these proportions were assumed to be 25%:75% as in [1].

### 3.4. Number of Screenings

Patients potentially having DRM are screened on admission and then once weekly. Patients with manifest DRM are only screened once on admission [19].

The number of screenings therefore depends on the proportions of patients with potential and manifest DRM and their expected LOS. Two studies showed that LOS in patients with DRM is longer than in well-nourished patients (11 vs. 7 days [16], 14 vs. 7.6 days [17]). Thus, it was assumed that LOS in patients without DRM was one week or less, and that LOS detected in DRM patients detected by NS was 12–14 days (1–2 weeks). Therefore, it was assumed that patients without DRM receive one NS, patients with potential DRM receive two NS, and patients with manifest DRM receive one.

### 3.5. Types and Proportions of NI

We distinguish between three types of NI: ONS, EN, and PN. In the study by Aeberhard et al., 59% of ND patients received ONS, 29% received EN, and 12% received PN [1]. All patients with potential DRM received ONS. Of the 59% of patients prescribed ONS, 25% were patients with potential DRM and 34% were patients with manifest DRM. The proportions of potential and manifest DRM differ in patients with NS. This implies that 45% with potential DRM will be prescribed ONS. In addition, of the 55% with manifest DRM, 25% will receive ONS, 21% will receive EN, and 9% will receive PN.

### 3.6. Reduction of LOS

Available data suggest that hospital LOS can be reduced by NI. However, it is difficult to quantify the amount. It seems that this effect is more likely to occur in patients with manifest DRM than with potential DRM and that it is more pronounced in connection with severe DRM [5,20,21,22]. Based on the findings of Elia et al. (−13.8% in relation to 22.5 hospital days) [3], Sriram et al. (−10% in relation to 6 days) [5] and Bally et al. (0%, calculated −3.2% of 13 days, not significant) [6], it was assumed here that NS will reduce LOS in patients with manifest DRM by 10%. This results in a reduction in LOS of 1.2 days. The same assumption is also made for ND patients.

### 3.7. DRG Changes

In ND, coding of a case of DRM led to a DRG change in 8.3% of all coded cases [1]. In patients with NS this proportion was substantially higher, amounting to 27% [16] and 15% [17]. Therefore, it was conservatively estimated to be 15%.

### 3.8. Increase in Cost-Weight

The average amount of additional revenue per case with a DRG change amounted to CHF 7564 [1]. Given a base rate of CHF 10,900, this corresponds to an average increase in the cost-weight of 0.694. However, in patients with NS, the average increase was clearly smaller (0.44) [16,17].

### 3.9. Ethics

This study was conducted in accordance with the ethical guidelines of the 1957 Declaration of Helsinki and approved by the Bernese Cantonal Ethics committee (BASEC ID 2017-00480), Bern, Switzerland.

## 4. Cost and Savings

### 4.1. Cost of NS

The costs of NS were calculated based on the time needed in minutes multiplied by the cost per minute of nursing staff. According to Wenger et al., the time needed to administer NS is about 5 min [19]. The costs per minute were calculated from an hourly rate (gross wage including employers’ contributions to social insurance, information University Hospital Bern) and amounted to CHF 3.93.

### 4.2. Costs per Patient with NI

Therapy costs per patient for NI patients with DRM included daily personnel and materials costs multiplied by the duration of NI in days. In the case of EN and PN, one-time costs per therapy were added (Table 2). These values were based on Aeberhard et al. [1] and updated for 2019.

For ONS, the time expended by staff members was 10 min for a nutritional therapist, 10 min for nursing staff, and 2 min for a physician; for EN it was 10 min for a nutritional therapist, 40 min for nursing staff, and 2 min for a physician; and for PN it was 12 min for a nutritional therapist, 70 min for nursing staff, and 2.4 min for physicians. The costs were CHF 41.40 per hour for a nutritional therapist, CHF 47.11 per hour for nursing staff, and CHF 78.24 per hour for physicians (information provided by the Bern University Hospital). Data on materials costs, duration of NI and one-time costs were obtained from Aeberhard et al. [1]. Thus, the costs per patient for NI were calculated as CHF 187.57 for ONS, CHF 842.96 for EN, and CHF 1557.84 for PN.

### 4.3. Savings per Prevented Inpatient Day

It was assumed that the reduction of LOS occurred at the end of the hospitalization period. The costs then were mainly related to accommodation, food, medical care, follow-up visits, hospital buildings and the like. Such LOS-dependent costs are also used for the remuneration of hospital services in DRG outliers. In such cases, in addition to the per-case rate, the hospital is reimbursed a daily rate for each day exceeding the upper trim point delimiting inlier LOS. These daily rates are based on cost-weights per day. The cost-weight per day was calculated at 0.126 as a weighted average across the SwissDRGs based on Aeberhard et al. [1]. This was multiplied by the base rate of the Bern University Hospital (CHF 10,900). Thus, the cost per prevented hospital day was estimated at CHF 1373.

### 4.4. Additional Revenue due to SwissDRG Change

The number of patients with SwissDRG changes were multiplied by the average increases in the cost-weights and the CHF 10,900 base rate of the Bern University Hospital.

## 5. Results

### 5.1. Effect of a Systematic NS

The patient flow and performance of systematic NS are summarized in Table 3.

For a systematic NS, 20,000 patients per year would have to be screened on hospital admission. Assuming a detection rate of 20%, 4000 patients (including potential and manifest DRM) would be detected. Of these, 45% (1800) will have potential DRM and 55% (2200) will have manifest DRM. Patients with potential DRM and a projected LOS of 12 days will experience a second instance of NS in the second week of their hospital stay. So, in total, 21,800 NS will be performed. The 1800 patients with potential DRM and an absolute 24.9% of the 2200 patients with manifest DRM (=997 patients) will receive ONS. Furthermore, 21.3% (=851 patients) will receive EN, and 8.8% (=352 patients) will receive PN. These NI will effect a reduction of LOS of 1.2 days per case in patients with manifest DRM. This will result in global savings of 2640 hospital days in 2200 patients.

In all 4000 DRM patients, DRM will be coded as a complication or comorbidity in the DRG system. This coding will cause a DRG change in 15% of all DRM coded cases (i.e., 600 patients). The resulting costs are summarized in Table 4.

A total of 21,800 NS will be performed per year at a cost of CHF 3.93 per unit of NS. Thus, the costs of NS will amount to CHF 85,583. The costs of NI are calculated in a similar way using the number of patients multiplied by the NI costs per patient. The costs for all 2797 patients with ONS (1800 with potential, 997 with manifest DRM) were calculated as CHF 524,694, the costs of the 851 patients with EN as CHF 717,076, and those of the 352 patients with PN as CHF 548,358. Overall, the costs of NI amount to CHF 1,790,128. Combined with the costs of NS, global costs amount to 1,875,711. These costs are balanced by savings of CHF 3,625,930 due to the reduction of LOS. Furthermore, additional revenue of CHF 2,877,600 results from DRG changes. So, after deduction of the costs, there is a net monetary gain of CHF 4,627,818 for the hospital.

### 5.2. Effects of ND, Treatment and Coding of DRM

Table A1 (Appendix A) shows the patient flow in the case of natural detection of DRM. In relation to the target population of 20,000 patients, the detection rate is 6.4%, which results in 1280 patients per year with DRM detected, treated and coded. Of these, 25% (320 patients) will be patients with potential DRM and 75% (960 patients) will be with manifest DRM. ONS will be provided to 755 patients (59%), EN to 371 patients (29%), and PN to 154 patients (12%).

It can be assumed that the average LOS in patients with naturally detected DRM would have been longer if they had not been treated for DRM and if NI had not reduced this LOS in the 960 patients with manifest DRM by 1.2 days. This amounts to a savings of 1152 hospital days. In 8.33% of the patients, i.e., in 107 cases, there is a DRG change.

Table A2 (Appendix A) shows the costs related to natural detection of DRM. The costs of NI amounts to CHF 141,652 for the treatment of 755 patients with ONS, CHF 312,906 for the 371 patients with EN, and CHF 239,284 for the 154 patients with PN. Total costs amount to CHF 693,842 per year. The savings due to a reduction of LOS amounted to CHF 1,582,224. The additional revenue resulting from SwissDRG changes amounts to CHF 806,579. The costs are overcompensated for by the savings and the additional revenue, resulting in an overall net savings of CHF 1,694,961.

### 5.3. Costs, Savings, and Additional Revenue Attributable to NS

As compared with the actual state of a natural detection, a systematic NS would generate additional costs of CHF 85,583 for screening and CHF 1,096,286 for NI, totaling CHF 1,181,869. These would be compensated for by savings of CHF 2,043,706 due to a reduction in LOS and by additional revenue of CHF 2,071,021 arising from coding and DRG changes (Table 5).

### 5.4. Staff Needed

Table A3 shows (Appendix A) the calculation of the staff needed, broken down by professional group. The total number of working hours and days was based on the time needed per day and the duration of the therapy in days (see Table 1) and on the number of patients undergoing therapy. Details are given in Table A4 (Appendix A). The number of positions was calculated using an average working day of 8.4 h, 220 working days per year and a fulltime position, and a productivity of 80%.

For a systematic NS, 4.09 positions for nutritional therapists, 9.27 positions for nursing staff, and 0.82 positions for physicians is needed. The treatment of naturally detected patients with DRM presently requires 1.35 positions for nutritional therapists, 3.6 positions for nursing staff, and 0.27 positions for physicians. Thus, the additional staff needed amounts to 2.75 positions for nutritional therapists, 5.66 positions for nursing staff and 0.55 positions for physicians.

### 5.5. Scenario Analyses

The results show that the savings and the additional revenue are each separately greater than the costs. Scenario analyses were performed based on few important assumptions to determine the minimum savings or additional revenues needed in order to cover only the costs. To find a best estimate for the reduction of LOS, the value of a saved hospital day, the proportion of DRG changes, and the average increases in the cost-weights, the following scenario analyses were performed:

Analysis 1, cost consequences in case the savings = 0, shows that even if no savings could be realized, NS could still achieve a net benefit through the additional revenue of CHF 1,001,889.

Analysis 2, cost consequences if additional revenue = 0, reveals that, even if no additional revenue were realized, NS would still lead to net savings of CHF 1,750,218.

Analysis 3, minimal additional revenue needed if savings = 0, shows that, in order to compensate for the costs, additional revenue of CHF 1,875,711 is needed. This could be achieved (1) by reducing the proportion of cases with changes in DRG attribution to 9.8% while keeping the increase in the cost-weight constant, or (2) by leaving the percentage of cases with changes in DRG attribution unchanged while increasing the average cost-weight by only 0.29.

Analysis 4 shows that a 5% reduction in LOS with a constant value per day would be sufficient to achieve the minimum required savings of CHF 1,875,711. On the other hand, if the reduction of LOS were left unchanged, a valuation of CHF 710 per saved hospital day would be sufficient.

## 6. Discussion

The introduction of a systematic NS would be accompanied by yearly costs of CHF 1.875 million. These costs would be compensated for by savings of CHF 3.635 million from a reduction in LOS and by additional revenue of CHF 2.877 million due to changes in documentation and coding of DRM. These numbers include the costs, savings and additional revenue produced by natural detection of DRM. These were estimated at CHF 0.693 million for the costs, CHF 1.582 million for the savings, and CHF 0.806 million for the additional revenue. Thus, the introduction of a systematic NS would generate additional costs of CHF 1.181 million that would be compensated for by additional savings of CHF 2.043 million and additional revenue of CHF 2.071 million.

These figures are based on projections using data from literature analyses, use of secondary statistics and data collected from the Bern University Hospital. It is notable that the savings per se are almost twice the costs, and the additional revenue alone is about 1.5 times the costs. These results are based on a few central assumptions about the data collected. The most important of these are discussed here.

The detection rate of NS was estimated to be 20%. Compared with data from the literature showing prevalence rates of 20–50% and even greater, this is a cautious assumption. The assumption regarding the size of the target population seems well justified, as only patients with an expected LOS of >3 days were considered. In estimating the expected LOS of patients with DRM, neither the average LOS (barely 6 days) of all patients from the Bern University Hospital nor the LOS (20 days) of the naturally detected patients in the study by Aeberhard et al. [1] can be considered representative. The expected LOS in patients with DRM (12 days) is plausible given that the average LOS of the target population is 10 days.

The costs of NS are very low compared with those of NI. The costs as well as the savings and the additional revenue are determined by the detection rate and the number of patients with DRM. Varying the assumptions relating to the detection rate will therefore result in a corresponding almost proportional variation in the difference between the costs and the savings and the additional revenue. This difference depends strongly on the assumptions regarding the savings and the additional revenue.

The projected savings are based on assumptions about the reduction of LOS due to NI. These are based on the results of meta-analyses, reviews and single studies. The literature is not consistent, but it shows evidence of or tendencies toward reduction of LOS, complication rates or rates of non-elective rehospitalizations [5,6,10,11,12,13]. However, for an economic assessment, available data on types and frequencies of avoided complications are not specific enough. Reductions in rehospitalizations are also difficult to evaluate because of varying follow-up periods. Therefore, it seems justified to use the reduction in LOS as a proxy for the many and various effects, with evidence of an improvement in clinical and economic outcomes due to NI.

For each one-day reduction in LOS, CHF 1371 was saved. This reflects the high costs associated with medical care in Switzerland and at university hospitals. A Danish study found the costs of outliers to be $224 per day in 2006. A Dutch study from the year 2005 estimated these costs at 476 €, and a US study reported costs of $1770 in 2018. The global average costs per hospital day at the Bern University Hospital amounted to CHF 2848 (information provided by the Federal Office of Public Health). So, valuing the LOS-dependent cost with per diem additional rates for DRG outliers seems justified.

The amount of additional revenues received is determined by the proportion of cases with DRG changes and the resulting increase in the average cost-weight in these cases. In the study by Aeberhard et al. [1], the proportion with changes in DRG attribution amounted to 8.3%. Compared with other studies in patients without NS, this is quite low. The average increase in the cost-weight was 0.694, a figure confirmed by other studies as well. In patients with NS, these numbers are not valid. The proportion of cases with changes in DRG attributions was assumed to be 15%. This is a conservative estimate and corresponds with the lower of two values from studies with NS (15% and 27%). The average increase in the cost-weight in these patients was lower, amounting to circa 0.44 in both studies, a figure, which was therefore applied in the current study.

## 7. Cost Effectiveness of Nutritional Therapy in the Post-Hospital or Community Setting: A Brief Statement

Prevalence of malnutrition has been studied less often in the community setting due to the lack of systematic or standardized screening programs, for example at healthcare institutions or in the offices of general practitioners. It is well established, however, that between 20% and 40% of patients admitted from the community setting to a hospital are already malnourished. Also, nutritional status frequently worsens during a hospital stay, which means that a large proportion of patients, particularly those older than 60 years, are discharged to a community setting or to other institutions with malnutrition. It is estimated that only around 10% of malnourished patients are in fact hospital patients, with the rest dwelling in a community or nursing home setting [23].

Few studies have addressed the costs of malnutrition and the economic impact of nutritional support in these settings [24]. Due to feasibility, the majority of studies investigating cost effectiveness of nutritional therapy are in-hospital analyses, but these only reflect a short period in terms of the patients’ needs for nutritional therapy. Malnourished patients, particularly those who are old, frequently need continued nutritional support. This is a challenge for the analysis of cost effectiveness, as the costs of management in one setting may be offset by greater cost savings in another setting, such as when patients are moving from one care setting to another and a more comprehensive perspective is needed. Not surprisingly, nutritional therapy is frequently discontinued after a hospital stay. Most studies that have assessed cost effectiveness of NI in the community-dwelling population are initiated at hospital discharge [25,26,27,28]. These frequently use oral nutritional supplements and are carried out for 3–6 months after a hospital stay. Studies of community-dwelling or nursing home residents more frequently address further types of nutritional support, such as dietary counseling, snacks between meals, or multi-component nutritional support.

Table A5 (Appendix A) gives an overview of the type and design of studies investigating the cost effectiveness of nutritional support following hospital discharge in the community-dwelling elderly or in nursing home residents. Despite the different methodologies used, nutritional therapy was generally found to be cost effective, with the higher costs incurred by nutritional support being ultimately associated with decreased use of health care resources and improved quality of life.

## 8. Conclusions

This is the first study to compare the costs of a systematic NS and subsequent adequate NI, with the combined effects of savings due to reduced LOS and additional revenue resulting from SwissDRG changes. The costs of the intervention could be separately compensated for by savings in the costs of basic treatment and by the additional revenue generated. NS with subsequent adequate NI is thus associated with high economic potential for the hospital. Moreover, the EFFORT trial was able to demonstrate an impressive effect of NI, with a significant reduction in mortality and severe complications as well as improvement of physical function and quality of life in medical inpatients [11]. These clinical benefits, in terms of savings in supplemental hospital costs, were not included in the current economic impact analysis. If they had been, this would have greatly increased the financial efficacy.

## Figures and Tables

**Table 1:** Overview of the input data.

Item	Both	Natural Detection	Nutritional Screening
Target population		Variable	20,000
Detection rate DRM		6.4%	20.0%
Proportions p DRM:m DRM		25% vs. 75%	45% vs. 55%
Number of nutritional screenings			No DRM, m DRM: 1; p DRM: 2
Nutritional interventions			
p DRM: ONS	100%	25%	45%
m DRM: ONS	45%	34%	25%
m DRM: EN	39%	29%	21%
m DRM: PN	16%	12%	9%
Reduction LOS in mDRM, days	1.2		
SwissDRG attribution change		8.3%	15.0%
Average increase cw		0.694	0.44

DRM: disease-related malnutrition; p DRM: potential DRM; m DRM: manifest DRM; ONS: oral nutritional supplements; EN: enteral nutrition; PN: parenteral nutrition; LOS: length of hospital stay; SwissDRG: Swiss diagnosis-related group, cw: cost-weight.

**Table 2:** Costs of nutritional interventions (NI) per patient and intervention.

	ONS	EN	PN
**Per day**			
Personnel costs, CHF	17.36	40.91	66.37
Materials costs, CHF	4.97	40.09	75.02
Personnel and materials costs, CHF	22.33	81.00	141.39
**Per therapy**			
Duration of therapy, days	8.4	10.3	9.6
Personnel and material costs, CHF	187.57	834.35	1357.36
One-time costs, CHF		8.61	200.48
Total therapy costs, CHF	187.57	842.96	1557.84

ONS: oral nutritional supplements; EN: enteral nutrition; PN: parenteral nutrition.

**Table 3:** Patient flow and resource use in connection with a systematic nutritional screening (NS).

Patients Flow and Performance	Proportion/Rate	Number
Target population	20,000	
Detection rate, of which:	20%	4000
- proportion p DRM	0.45	1800
- proportion m DRM	0.55	2200
Number of systematic nutritional screenings		
- On hospital admission	1	20,000
- Weekly in cases with p DRM	1	1800
Total screenings		21,800
Nutritional interventions		
p DRM		
- ONS	45.0%	1800
m DRM		
- ONS	24.9%	997
- EN	21.3%	851
- PN	8.8%	352
Total nutritional interventions	100.0%	4000
Saved hospital days	Per Patient	Total
expected LOS	12	
reduction %	10%	
Reduction LOS, days	1.2	2640
Swiss DRG changes	Detected Cases	
Changes in DRG attribution	0.15	600

DRM: disease-related malnutrition; p DRM: potential DRM; m DRM: manifest DRM; ONS: oral nutritional supplements; EN: enteral nutrition; PN: parenteral nutrition; LOS: length of hospital stay; DRG: diagnosis-related groups.

**Table 4:** Costs resulting from a systematic NS.

	Number	Value	Costs
Costs			
Systematic nutritional screening	21,800	3.93	85,583
Nutritional interventions			
ONS	2797	187.57	524,694
EN	851	842.96	717,076
PN	352	1557.84	548,358
Total nutritional interventions	4000		1,790,128
Total costs			1,875,711
Savings (LOS reduction) (−)	2640	1373.46	−3,625,930
Additional revenue (SwissDRG) (−)	600	4796.00	−2,877,600
Net effect			−4,627,818

Costs are indicated with positive (+), savings and additional revenue with negative (−) prefix. DRM: disease-related malnutrition; p DRM: potential DRM; m DRM: manifest DRM; ONS: oral nutritional supplements; EN: enteral nutrition; PN: parenteral nutrition.

**Table 5:** Net monetary effects of the introduction of a systematic NS.

	Nutritional	Natural	Extra Costs/
	Screening	Detection	Savings NS
Costs			
Systematic screening	85,583		85,583
Nutritional interventions	1,790,128	693,842	1,096,286
Total costs	1,875,711	693,842	1,181,869
Savings (reduction LOS) (−)	−3,625,930	−1,582,224	−2,043,706
Additional revenue (SwissDRG) (−)	−2,877,600	−806,579	−2,071,021
Net effect	−4,627,818	−1,694,961	−2,932,858

## Appendix A

**Table A1:** Patient flow and resource use in connection with ND.

Patients Flow and Performance	Proportion/Rate	Number
Target population	20,000	
	Proportion/Rate	*n*
Detection rate, of which:	6.4%	1280
proportion p DRM	0.25	320
proportion m DRM	0.75	960
Nutritional interventions		
	Proportion	*n*
ONS	59%	755
EN	29%	371
PN	12%	154
Saved hospital days	Per Patient	total
Reduction in LOS, days	1.2	1152
SwissDRG changes	Detected Cases	
	Proportion	*n*
Changes in DRG attribution	0.0833	107

DRM: disease-related malnutrition; p DRM: potential DRM; m DRM: manifest DRM; ONS: oral nutritional supplements; EN: enteral nutrition; PN: parenteral nutrition; LOS: length of hospital stay; DRG: diagnosis-related groups.

**Table A2:** Consequences of the costs of ND.

	Number	Value	Costs
Costs nutritional Interventions			
ONS	755	187.57	141,652
EN	371	842.96	312,906
PN	154	1557.84	239,284
Total costs	1280		693,842
Savings (reduction LOS) (−)	1152	1373.46	−1582,224
Additional revenue (SwissDRG) (−)	107	7564.70	−806,579
Net effect			−1,694,961

Costs are indicated with positive (+), savings and additional revenue with negative (−) prefix. DRM: disease-related malnutrition; p DRM: potential DRM; m DRM: manifest DRM; ONS: oral nutritional supplements; EN: enteral nutrition; PN: parenteral nutrition.

**Table A3:** Hours, workdays and positions needed for NI in the case of systematic NS, ND and additional needs for systematic NS.

	Nutritional Therapy	Nursing	Physician
Systematic nutritional screening			
Hours total	6052	13,700	1210
Days total	721	1631	144
Positions needed	4.09	9.27	0.82
Natural Detection			
Hours total	1989	5327	398
Days total	237	634	47
Positions needed	1.35	3.60	0.27
Additional needs for systematic nutritional screening			
Hours total	4063	8373	813
Days total	484	997	97
Positions needed	2.75	5.66	0.55

**Table A4:** Staff time per nutritional intervention and professional group.

		Dietician	Nursing Staff	Physician
ONS				
Duration of therapy, days	8.4			
Minutes/patient/day		10	10	2
Minutes/patient		84	84	16.8
N patients NS	2797			
Minutes for NS		234,976	234,976	46,995
N patients ND	755			
Minutes for ND		63,437	63,437	12,687
EN				
Duration of therapy, days	10.3			
Minutes/patient/day		10	40	2
Minutes/patient		103	412	20.6
N patients NS	851			
Minutes for NS		87,619	350,475	17,524
N patients ND	371			
Minutes for ND		38,234	152,934	7647
PN				
Duration of therapy, days	9.6			
Minutes/patient/day		12	70	2.4
Minutes/patient		115.2	672	23.04
N patients NS	352			
Minutes for NS		40,550	236,544	8110
N patients ND	154			
Minutes for ND		17,695	103,219	3539

DRM: disease-related malnutrition; p DRM: potential DRM; m DRM: manifest DRM; ONS: oral nutritional supplements; EN: enteral nutrition; PN: parenteral nutrition.

**Table A5:** Studies addressing the economic impact of nutritional support after hospital discharge in the community or nursing home setting.

Nutritional Support After Hospital Discharge				
Author	Population	Type of Study	Cost Analysis	Results
Edington et al., 2004 [29] United Kingdom	mixed malnourished ≥ 65 y, (*n* = 100)	RCT intervention ONS for 8 weeks, 24 weeks follow-up	Cost-effectiveness analysis, direct and indirect costs	No difference regarding quality of life, post-hospital health care resource use or cost
Norman et al., 2011 [25] Germany	gastrointestinal disease, malnourished (*n* = 114)	RCT intervention Dietary counseling at discharge and high-protein ONS for months vs. dietary counseling at discharge	Cost-effectiveness analysis, direct costs of nutritional support	Intervention patients: increase in quality of life after 3 months ICER: EUR9497–12099/QALY
Neelemaat et al., 2012 [26] The Nederlands	mixed, malnourished ≥ 60 y (*n* = 210)	RCT: ONS, dietary counseling, vitamin D for 3 months after hospital discharge vs. usual care	Cost-effectiveness analysis, direct and indirect costs	No significant difference in QALYs at 3 months follow-up, intervention group: improvement in functional limitations, EUR618/functional limitation improvement (0.95 probability the intervention is cost effective)
Zhong et al., 2016 [27] United States of America	Mixed, malnourished ≥ 65 y (*n* = 622)	RCT: high-protein ONS, enriched b-hydroxy-b-methylbutyrate for 3 months after hospital discharge compared to placebo	Cost-effectiveness analysis, direct and indirect costs	Intervention group: increase in quality of life ICER: USD 33,818/QALY lifetime ICER: USD 524/LY
Nutritional support in the community or nursing home setting				
Author	Population	Type of study	Cost analysis	Results
Arnaud-Battandier et al., 2004 [30] France	Malnourished ≥ 70 y community or nursing home residents (*n* = 287)	prospective, cohort study of patients from 2 groups of physicians (high vs. low ONS prescription rate), 12-month follow-up	Comparison of direct costs	Higher costs of ONS in intervention group (EUR M, but lower costs of medical care: hospital admissions (EUR1631 vs. EUR 2203) and medical visits (EUR 299 vs. EUR 462)
Lorefält et al., 2011 [31] Sweden	nursing home residents, ≥ 65 y (*n* = 109)	Prospective cohort study of nutrition education and care (individualized meals) for 3 months	Comparison of direct costs	Higher costs in intervention group (EUR 830 vs. EUR 760 for nutritional support, EUR 652 vs. EUR 402 for education program)
Freijer et al., 2013 [32] The Nederlands	malnourished ≥ 65 y; community or nursing home residents (*n* = 720,223)	Health economic evaluation of published studies	Budget impact analysis	Annual cost savings of EUR 11.62 million due to intervention with ONS
Schilp et al., 2014 [28] The Nederlands	malnourished ≥ 65 y; community-dwelling old (*n* = 146)	RCT, dietary counseling vs. usual care	Cost-effectiveness analysis	No differences regarding gain in weight, QALY or costs
Simmons et al., 2015 [33] United States of America	Malnourished/at risk ≥ 65 y, nursing home residents (*n* = 154)	3-arm RCT: ONS vs. in-between snacks vs. usual care for 6 months	Cost-effectiveness analysis	No change in body weight, intervention costs per person per day; ONS group 2.54 and snack group 3.85; ICER: 103 kcal/USD in ONS group vs. 79 kcal/USD in snack group
van der Pols-Vijlbrief et al., 2017 [34] The Nederlands	community-dwelling older adults receiving home care with or at risk of malnutrition ≥65 y (*n* = 155)	RCT, multifactorial personalized intervention for 6 months	Cost-effectiveness analysis	No differences regarding gain in weight, functional status, QALY or costs
Elia et al., 2018 [35] United Kingdom	Malnourished, nursing home residents ≥65 y (*n* = 104)	RCT, comparing ONS versus dietary advice for 3 months	Cost effectiveness analysis with direct and indirect costs	Intervention group improved quality of life: ICER: GBP 10,961/QALY

ONS: oral nutritional supplements; QALY: quality of life adjusted life year; ICER: incremental cost-effectiveness ratio.
